# Renal histomorphology in dogs with pyometra and control dogs, and long term clinical outcome with respect to signs of kidney disease

**DOI:** 10.1186/1751-0147-49-13

**Published:** 2007-05-04

**Authors:** Reidun Heiene, Veronica Kristiansen, Jon Teige, Johan Høgset Jansen

**Affiliations:** 1 Department of Companion Animal Clinical Sciences, Norwegian School of Veterinary Science, PO Box 8146 Dep., N-0033 Oslo, Norway; 2 Department of Basic Science and Aquatic Medicine, Norwegian School of Veterinary Science, PO Box 8146 Dep., N-0033 Oslo, Norway

## Abstract

**Background:**

Age-related changes in renal histomorphology are described, while the presence of glomerulonephritis in dogs with pyometra is controversial in current literature.

**Methods:**

Dogs with pyometra were examined retrospectively for evidence of secondary renal damage and persisting renal disease through two retrospective studies. In Study 1, light microscopic lesions of renal tissue were graded and compared in nineteen dogs with pyometra and thirteen age-matched control bitches. In Study 2, forty-one owners of dogs with pyometra were interviewed approximately 8 years after surgery for evidence ofclinical signs of renal failure in order to document causes of death/euthanasia.

**Results:**

Interstitial inflammation and tubular atrophy were more pronounced in dogs with pyometra than in the control animals. Glomerular lesions classified as glomerular sclerosis were present in both groups. No unequivocal light microscopic features of glomerulonephritis were observed in bitches in any of the groups.

Two bitches severely proteinuric at the time of surgery had developed end stage renal disease within 3 years. In five of the bitches polyuria persisted after surgery. Most bitches did not show signs of kidney disease at the time of death/euthanasia.

**Conclusion:**

Tubulointerstitial inflammation was observed, but glomerular damage beyond age-related changes could not be demonstrated by light microscopy in the dogs with pyometra. However, severe proteinuria after surgery may predispose to development of renal failure.

## Background

In both human [1] and veterinary nephrology [2,3]., proteinuria has been shown to contribute substantially to the development of end stage renal disease. Clinical intervention by drug therapy is indicated to protect renal function [4].

Proteinuria is the hallmark of glomerular disease. Polyuria, polydipsia (PU/PD), proteinuria and azotemia are common features of canine pyometra [5-10]. Because polyuria/polydipsia usually disappear after treatment, the accompanying renal lesions are described as temporary. The renal pathology and long term clinical outcome in dogs with pyometra is poorly defined. Controversy exists as to whether pathological findings indicative of a tubulointerstitial nephritis and glomerulonephritis are more severe in dogs with pyometra than in healthy dogs ofcomparable ages.

In numerous textbooks, an immune complex mediated glomerulonephritis has been suggested as secondary to pyometra, although data is scarce and inconclusive [11-13]. Obel et al [14] examined renal tissue from 27 dogs with pyometra by light and electron microscopy and immunohistochemical methods. Tubulointerstitial and glomerular lesions were described and discussed in the context of an immune mediated glomerulonephritis. The results were compared to findings in a control group, which included five bitches between 1 to 6 years of age. Dogs with pyometra usually are older. Sandholm et al [15] described glomerular deposits following immunohistochemical staining of tissue from 12 dogs with pyometra and also interpreted the results in the context of immune mediated glomerular disease, although the number and age of dogs in the control group was not given.

In a larger prospective study, Stone et al [16] compared renal biopsies from 27 dogs with pyometra to biopsies from 12 age-matched control dogs using light microscopy, electron microscopy and immunohistochemistry. The prevalence and severity of glomerular and tubular morphological changes detected by light microscopy were similar in dogs with pyometra and control animals. Although the control dogs had a higher percentage of electron dense deposits than the dogs with pyometra, the overall evaluation of the renal tissue indicated no histological differences between the groups. To our knowledge, that is the only study which has examined renal lesions in dogs with pyometra and control dogs of comparable age.

Age-related changes in renal histomorphology and kidney function are well known from human medicine. Decline in renal function in aging individuals may represent a pathological process or may be intrinsic to the normal ageing process [17,18]. Prior studies have described glomerulosclerotic and glomerulonephritic lesions in old dogs with renal disease, with no known renal disease, and with diseases unrelated to renal function [19-23]. In a group of 230 Kansas greyhounds at different ages, 4% had macroscopic renal abnormalities, and 24.8% of the dogs had microscopic renal lesions, including glomerular changes [23]. Age related changes in renal tissue are also reported from a colony of beagle dogs [24].

Heiene et al [9] graded renal tubular changes in 55 dogs with pyometra (mean age 7.6 years). The dogs were monitored daily for two weeks and re-evaluated at 2–4 months after ovariohysterectomy. A substantial number of dogs had proteinuria at 2–4 months. In a different group of 6 dogs with pyometra, reversal of proteinuria was sometimes, but not always, observed after surgery [25].

The aims of the current studies were (Study 1) to compare renal histomorphology in dogs with pyometra from the above original study [9] retrospectively to control dogs of comparable age, and (Study 2) to report the results of aquestionnaire to dog owners regarding clinical outcome approximately 8 years after surgery.

## Methods

### Study 1 – Histopathological examination and evaluation

Nineteen dogs with pyometra (mean age 8.7 years of age; range 7–14) and 13 control dogs (mean age 9.7 years of age; range 7–13) were included in the present study. They were randomly the 19 first dogs above the age of 7 years of age included in the original study [9]. The number of dogs was selected in order to study a pyometra group of comparable size to the available control group.

The term pyometra in this paper includes the disease complex characterized by typical clinical signs with or without vaginal discharge and cystic endometrial hyperplasia with endometritis [26].

Thirteen control dogs were selected from autopsy material submitted to the Section of Pathology, Department of Basic Sciences and Aquatic Medicine, Norwegian School of Veterinary Science, during the previous 5 years, where renal tissues were routinely available from all cases. The evaluation of whether or not the dog fulfilled the inclusion criteriae was based upon the clinical and pathological patient journal. Autopsy material was obtained from control bitches that were included on the basis of complete medical records and absence of chronic medical diseases including PU/PD and proteinuria.

Table 1 provides clinical data on factors such as the presence of urinary tract infection, level of proteinuria on the day of surgery and 2–4 months later, and level of leucocytosis, which may have been of relevance to the biopsy results for the 19 dogs with pyometra in the original study.

**Table 1:** Selected clinical data from the dogs with pyometra: Level of leucocytosis, presence of urinary tract infection as confirmed by culture of cysteocentesis urine during surgery, and level of urine protein to creatinine ratio on the day of surgery and 2–4 months later

**Dog no.**	**WBC in blood X10e9/L (6.0–18.0 X10e9/L†)**	**Urinary Tract Infection**	**Urinary protein:creatinine ratio on the day of surgery (0,5)**	**Urinary protein:creatinine ratio 2–4 months after surgery**
P1	47,2	-	0	NA
P2	11,2	-	0	NA
P3	53,4	+	2,7	0
P4	26,6	-	9	0
P5	15,0	+	5,6	NA
P6	28,2	-	2,9	NA
P7	20,1	-	6,25	0
P8	21,0	-	0	NA
P9	17,5	+	0	NA
P10	19,6	-	0	0,7
P11	65,3	-	NA	10,3*
P12	NA	+	1,3	NA
P13	33,2	+	1	0
P14	27,7	-	0	0
P15	31,1	+	1,1	NA
P16	8,0	+	2	16,4
P17	54,7	+	3,6	0
P18	15,5	-	0,9	0,8
P19	6,6	+	0,8	0,6
Px	12,3	-	4	13

† reference values in healthy dogs at the Central Laboratory, Norvegian Schoool of Veterinary Science. NA = not available.* Urine protein creatinine ratio on day 9 after surgery. Reference values are given in parenthesis. Due to poor owner compliance the dog was lost to follow up and not available for the questionnaire. P16 = Dog dead from renal failure (questionnaire/clinicopathological data) Px = Dog dead from renal failure (questionnaire/clinicopathological data; this dog was not in the histology study)

Renal biopsies of dogs with pyometra were obtained during ovariohysterectomy using theBard^® ^Biopty-Cut^® ^device (C.R. Bard Inc., Covington, GA, USA) with 14G (1,2 mm × 19 mm) biopsy needles, and were processed for light microscopy. All biopsies contained 5 glomeruli or more (no. of glomeruli: min. 5, max. 38, median 17). Renal wedge specimens from control dogs were taken at autopsy. In order to avoid a bias of interpretation caused by autolytic changes in post-mortem control specimens, the pathological evaluation primarily focused upon evaluation of tissue cellularity and the extent of extracellular matrix deposition.

Renal tissue (biopsies or autopsy wedge specimens) were fixed by immersion in phosphate-buffered 4% formaldehyde, dehydrated in graded ethanols and embedded in paraffin. Four-micrometer thick sections were stained with hematoxylin-eosin (HE), elastin van Gieson (EvG), periodic acid-Schiff (PAS), periodic acid silver methenamine (Jones; PASM), andMasson's trichrome stain. The renal specimens were evaluated with respect to the histomorphology of each of the four major compartments of the renal parenchyma; glomeruli, tubules, interstitium and vessels. The severity of lesions in each compartment, including the severity of individual lesions as well as the degree of distribution of changes, was assessed by a semiquantitative method, from 0 through 3 (0 = normal, 1 = mild, 2 = moderate, 3 = severe)[27] Because of variation in post-mortem changes in specimens from control dogs, comparison of the condition of the renal tubules between dogs in the two groups was assessed by evaluation of the thickness of tubular basement laminae and the regularity of the tubular circumference. Microphotographs were captured using a Nikon DS-5M-U1 digital camera (Nikon Instech CO, Kawasaki, Kanagawa, Japan) mounted on a Leitz Laborlux K microscope (Leica Microsystems Wetzlar GmbH, Wetzlar, Germany).

### Study 2 – Owner questionnaire

Owners ofthe 55 dogs in the original study were contacted for the Study 2 follow-up detailed questionnaire; 41 owners responded. Questionnaires were completed by telephone, including

1) the dogs' drinking behaviour after surgery, and if increased drinking was persistent during the rest of the dogs life, 2) whether the cause of death was known (in which case confirmation was sought from the local veterinarian) 3) potential signs or diagnosis of renal failure after surgery.

The cause of euthanasia was classified as not renal failure when the cause of death was described in specific clinical terms, e.g. malignant histiocytosis, or when clinical signs could not be confused with renal failure, e.g. hip dysplasia, so that renal failure was unlikely. The cause of euthanasia was classified as unknown when the cause of death or euthanasia was not known. When a dog was reported dead from renal disease or unspecified disease, the patients clinical details were inspected to confirm a diagnosis at the time of euthanasia.

Although statistical analysis was not performed for individual clinicopathological parameters, there was no apparent correlation between the renal parameters reported in the original study and clinical outcome 8 years later. However, in the dogs dead from renal disease, the original classification of tubular lesions, glomerular filtration rate, and the levels of leucocytosis, enzymuria, proteinuria and bacteruria were re-evalutated for potential significance, by referring to original patients clinical details.

## Results

### Study 1 – Histopathological examination and evaluation

**Figure 1:**
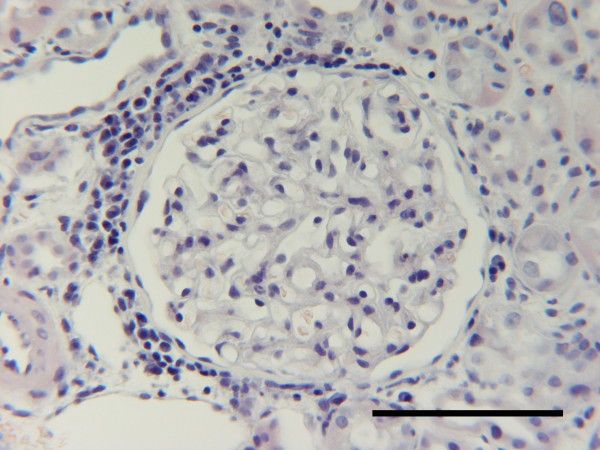
Kidney, dog. Periglomerular interstitial infiltration of plasma-lymphocytic cells following pyometra. Tissue from P4, Table 2. HE. Bar = 100 μm.

**Figure 2:**
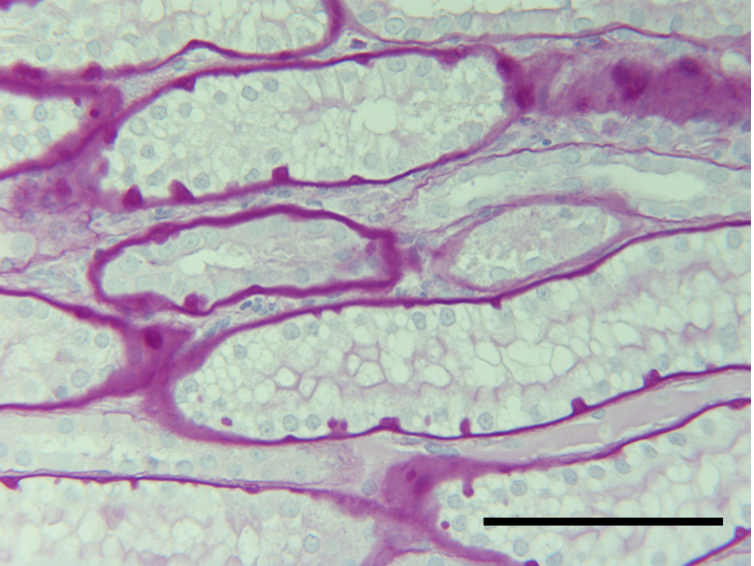
Kidney, dog. Thickened peritubular basal laminae with numerous nodular protrusions along the subepithelial side in atrophic straight juxtamedullary tubular segments in a dog with pyometra. Tissue from P17, Table 2. PAS. Bar = 100 μm.

**Figure 3:**
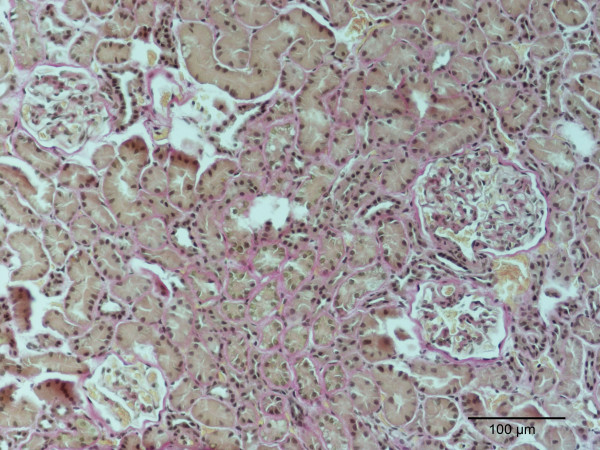
Kidney, dog. Diffuse interstitial and glomerular fibrosis in a dog with pyometra, P10, Table 2. Van Giesson. Bar = 100 μm.

The classification of renal lesions in individual dogs is presented in Table 2. Infiltration of the cortical interstitial tissue with predominantly mononuclear inflammatory cells consistent with plasma cells and lymphocytes was observed in 10 (52.6%) of the dogs with pyometra as compared to only 2 (12.5%) control dogs. However, polymorphonuclear leucocytes were observed as the main interstitial inflammatory cell type in two of the dogs with the pyometra. A conspicuous periglomerular distribution of the plasma-lymphocytic interstitial infiltrates was observed consistently in dogs with pyometra (Fig. 1). Mild to severe tubular atrophy was prominent morphological feature in 12 (63.2%) of dogs with pyometra, compared to 2 (12.5%) of the control dogs (Fig. 2). Excessive amounts of interstitial collagen indicative of interstitial fibrosis was observed in 16 (84.2%) of the dogs with pyometra, but was present in only 4 (25%) control dogs (Fig. 3).

**Table 2:** Glomerular, tubular and intestitial lesions in 19 dogs with pyometra (P1–P19) and 13 control dogs (C1 – C13)

**Dog no.**	**Available no. of glomeruli**	**Glomerular lesions**		**Tubular lesions**	**Interstitial lesions**	
		**Sclerosis**	**Fibrosis**	**Tubular atrophy**	**Inflammatory cellular infiltration**	**Fibrosis**

P1	10	1	1	1	1	1
P2	22	0	0	2	0	0
P3	17	0	0	0	1	1
P4	14	0	1	1	1	1
P5	19	0	0	0	1	1
P6	17	0	0	0*	1	0
P7	34	0	0	0	1**	1
P8	29	0	0	1	1	1
P9	10	0	0	0	0	1
P10	21	2	2	3	0	3
P11	18	1	1	0	1	1
P12	10	1	0	2	0	1
P13	8	0	0	2	0	1
P14	37	1	0	0	0	0
P15	21	2	1	2	1	1
P16	6	1	1	2	0	2
P17	38	0	1	1	1**	1
P18	5	0	1	1	0	1
P19	5	0	0	1	0	1***

C1	a.s.	0	1	0	0	0
C2	a.s.	1	2	0	0	1
C3	a.s.	0	1	0*	0	0
C4	a.s.	1	2	0	0	0
C5	a.s.	0	1	0*	0	0
C6	a.s.	0	2	2	2	3
C7	a.s.	1	0	0	0	0
C8	a.s.	2^†^	1	1	0	1
C9	a.s.	1	0	0	0	0
C10	a.s.	1	0	0	0	0
C11	a.s.	0	1	0	0	1
C12	a.s.	1	1	0	0	0
C13	a.s.	0	0	0	0	0

a.s.: autopsy specimen; * rich amounts of intraepithelial pigment (lipofuschin) in proximal tubules; **polymorphoduclear cells PMN; *** fibrous thickening of the Bowman's capsule; ^†^: hyalinisation of arterioles

The severity of lesions in each compartment, including the severity of individual lesions as well as the degree of distribution of changes, was assessed by a semiquantitative method, from 0 through 3 (0 = normal, 1 = mild, 2 = moderate, 3 = severe)

**Figure 4:**
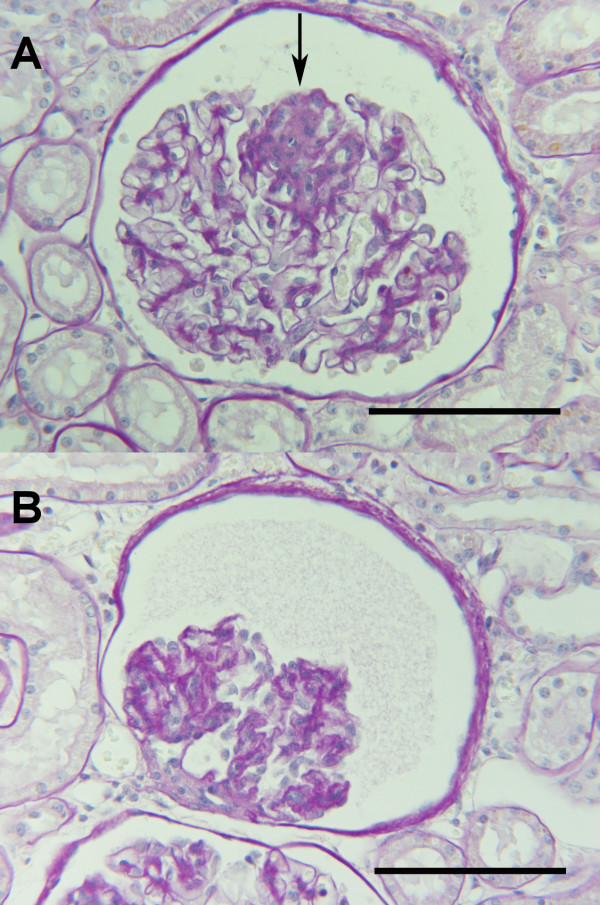
**Kidney, dog**. a. Segmental sclerotic glomerular lesion (arrow) in a control dog; C7, Table 2. b. Glomerulus from a control dog; C7, Table 2, revealing global advanced stage sclerosis.

**Figure 5:**
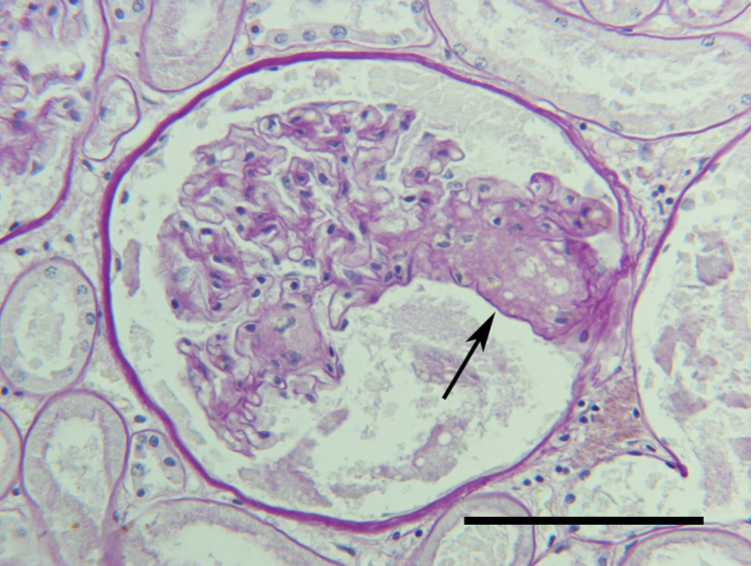
Kidney, dog. Segmental glomerular sclerosis in a control dog; C10, Table 2, showing a hyaline nodular mass containing numerous small lipid vacuoles (arrow). PAS. Bar = 100 μm.

**Figure 6:**
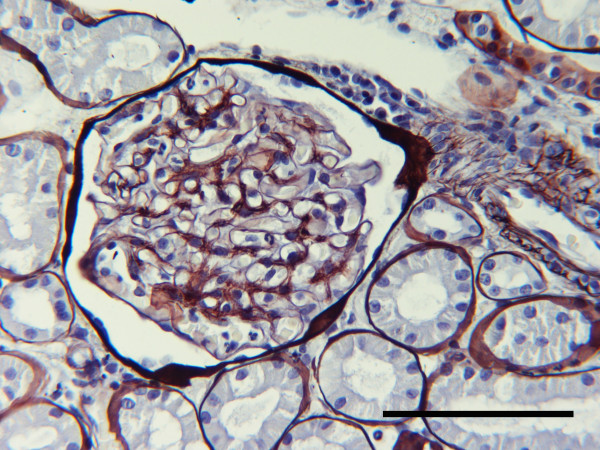
Kidney, dog. Non-sclerotic glomerulus with several intracapillary PMN's and periglomerular infiltration of plasma-lymphocytic cells in a dog following pyometra; P17, Table 2. PASM. Bar = 100 μm.

No unequivocal signs of glomerulonephritis were observed in any of the renal specimens in either of the two groups. The glomerular basement membranes (GBM) in sections stained with PAS and PASM had normal thickness and were generally devoid of any irregularities. Glomerular lesions included minor segmental increments in mesangial matrix staining positive with PAS, slight and focally distributed mesangial cell proliferation, and varying degrees of collapse and condensation of the GBM. These lesions were considered consistent with segmental glomerular sclerosis. Segmental (Fig. 4a) or global (Fig. 4b) glomerular sclerosis could be found among groups of glomeruli appearing normal (focal distribution). Glomerular sclerosis was observed in 7 (36.8%) of the dogs with pyometra and 9 (56.3%) of the control dogs. Glomerular fibrosis, as defined by the presence of collagen fibers staining red with the van Giesson stain and differentiated from sclerosis by not staining with the PAS reagent or PASM, was observed in 8 (42.1%) dogs with pyometra and 10 (62.5%) of the control dogs. Glomerular fibrosis was observed in 9 dogs devoid of evidence of glomerular sclerosis. In some control dogs, discrete mesangial areas containing increased amounts of matrix with a foamy appearance were present in sclerotic glomerular segments (Fig 5). In some dogs with pyometra, several neutrophils could be observed in the luminae of glomerular capillaries of non-sclerotic glomeruli (Fig. 6). Salient hyalinisation of the renal cortical arterioles and concomitant tubular atrophy was present in one control case (C8, Table 2).

### Study 2 – Owner questionnaire

Among the 41 dogs available for follow up by the questionnaire, four dogs were still alive. Five dogs had permanent polyuria and polydipsia after surgery; 36 dogs showed no signs of renal failure. Two dogs were dead from renal disease confirmed by blood samples and autopsy. In 11 dogs the terminal disease was not well described or known, and renal disease, although unlikely, could not be fully excluded. Twenty-four dogs were dead from conditions apparently not related to renal failure.

Blood, urine and biopsy samples from the two dogs with renal failure confirmed markedly elevated serum urea and creatinine levels, and renal lesions consistent with end stage renal disease. Both of these dogs had had severe proteinuria in the original study, with urinary protein:creatinine ratios above 10 during the re-check visit (Table 1). One of them was among the 5 dogs with permanent PU/PD after surgery;the other developed PU/PD a few weeks before uremic crisis 3 years after surgery.

## Discussion

The most prominent morphological difference between two groups was the interstitial inflammatory infiltrates prevalent in dogs with pyometra. These plasma-lymphocytic interstitial infiltrates, often with a periglomerular location, were accompanied by a higher prevalence of interstitial fibrosis and tubular atrophy in dogs with pyometra (Figures 1 and 3). These observations are in accordance with previous reports of renal lesions in dogs with pyometra [14].

Earlier studies in dogs with pyometra [14] interpreted glomerular lesions to be membranous or mixed membranous and proliferative glomerulonephritis. More recent studies and glomerular disease classification, suggest that this type of glomerular lesion, also observed in the present study, are consistent with glomerular sclerosis. The incidence of membranoproliferative glomerulonephritis has likely been overestimated in older veterinary literature[28]. Stone et al [16] reported a high prevalence of mild tubulointerstitial nephritis in dogs with pyometra, but only minor damage to renal tubules and few specific glomerular lesions. Firm conclusions should not be drawn on the basis of the limited amount of data presented here; yet the notion that glomerulonephritis is prevalent in dogs with pyometra is not supported by findings of the current study.

The occurrence of glomerular sclerosis and glomerular fibrosis in both study groups indicate that glomerular sclerosis and fibrosis may be a coincidental finding in the aged dog affected by pyometra. This assumption is supported by previous reports on renal lesions in ageing dogs [23,24].

The glomerular sclerotic lesions observed in the present study were often distributed unevenly throughout the renal cortices, and frequently showed segmental distribution within individual glomeruli. The observed lesions resembled secondary forms of focal segmental glomerular sclerosis (FSGS) in humans. In the human nephrology, FSGS is also sometimes observed as a component of age related phenomena [29].

Human FSGS is associated with clinical manifestation of proteinuria or the nephrotic syndrome. However, in the present study, the magnitude of proteinuria did not seem to correlate with the degree of glomerular sclerosis observed in the biopsies (Table 1). Obel et al [14] suggested hyaline droplet degeneration of proximal tubules as an indicator of glomerular protein leakage, but could not correlate tubular lesions to the degree of glomerular damage. In the present study, light microscopic glomerular lesions were found similar to those described in the latter report, suggesting that glomerular sclerosis may be associated less with proteinuria in dogsthan in people. However, veterinary nephropathology is yet to provide a detailed classification system for glomerular disease with clinico-pathological correlates. The relationship between glomerular sclerosis and proteinuria in the dog remains to be defined.

Increasing amounts of interstitial fibrosis and age-associated glomerular sclerosis has been described in people [30,31]. Kappel and Olsen [30] reported that in humans, the percentage of sclerotic glomeruli was 0–1% in people below 40, but increases to 30% in persons more than 80 years of age. The sclerotic glomeruli atrophy and eventually disappear with age [32].

Renal lesions also have been demonstrated in aged dogs [19,21,23]. Age related changes are relevant for interpretation of pathological processes in the kidney in a toxicological or disease context [24]. The higher age of the control dogs compared to the dogs with pyometra may have influenced the prevalence of glomerular sclerosis observed in the present study.

Given the high prevalence of proteinuria in dogs with pyometra [9], a significant glomerulopathy is likely to be present. Light microscopy alone does not provide opportunity to evaluate the detailed nature of the glomerular filter. In human nephropathology additional electron microscopy and immunofluorescence studies have provided additional data for the understanding of glomerulopathies. These modalities will be critical to future studies in veterinary nephrology [28], but were not available in this study. Proteinuria in the dogs of this study (Table 1) may not have been solely due to glomerular loss, as some of them also had bacterial cystitis. However, only a small proportion of dogs with bacterial cystitis have high urinary protein creatinine ratios [33].

Future studies should attempt to define the nature of the acute glomerular damage to the glomerular filter, in order to optimize patient care after pyometra. Since the early 1990's reports have described reduced proteinuria and a "renoprotective" effect of angiotensin converting enzyme (ACE) inhibition and strict blood pressure control in renal disease in humans [34]. A similar effect also has been described in dogs with glomerulonephritis [35]. Blood pressure control and ACE inhibition has become routine in canine nephrology. One recent study in humans demonstrated that proteinuria is a strong independent predictor of end stage renal disease in a mass screening setting [1]. Some dogs with pyometra and severe proteinuria progress to fulminant renal failure, as was observed in the two dogs with documented renal disease as the cause for euthanasia in the present study. This finding illustrates the importance of post-surgical follow up of proteinuria in dogs with pyometra. Heiene et al [25] reported progression to renal failure in one proteinuric dog out of 6 dogs with pyometra, in spite of close follow up and treatment.

The inherent subjectivity of questionnaire results, as well as semiquantitative histological grading, allows for descriptive presentation of the material, rather than statistical analysis. More accurate results could be expected if a thorough clinical examination and questions to the owner had been performed soon after the original study. More optimal data from control dogs would have been prospective material, where fresh kidney tissue and a detailed clinical history was available. Future studies should take such considerations into account and also include electron microscopy and immunohistochemistry.

Nevertheless, our data are not consistent with the commonly held notion that pyometra leads to an immune-mediated glomerulonephritis. Current literature is equivocal on this point. The two studies indicating immune mediated glomerulonephritis do not include an age matched control group [14] or no control group [15]. Immune deposits in glomeruli of healthy individuals are documented in pigs [36] and in humans [37]. Glomerular immune deposits are documented in dogs without known kidney disease; predominantly in old dogs [22]. In one controlled study [16], pyometra related changes in the kidney were similar in severity to age related changes in healthy dogs, as evaluated by light microscopy, electron microscopy and immunohistochemistry. Although our data does not include immunohistochemistry; the light microscopic changes observed in our study appear to support the conclusions from the latter study.

## Conclusion

histopathological examination and evaluation revealed tubular and interstitial lesions in dogs with pyometra, but histological features specific for glomeruonephritis were not prominent. Glomerular sclerosis was prevalent in dogs with pyometra and in control dogs. The questionnaire did not reveal clinical signs of kidney disease after surgery in most dogs with pyometra; although PU/PD was observed in five dogs and two dogs died from renal disease.

## Competing interests

The author(s) declare that they have no competing interests.
